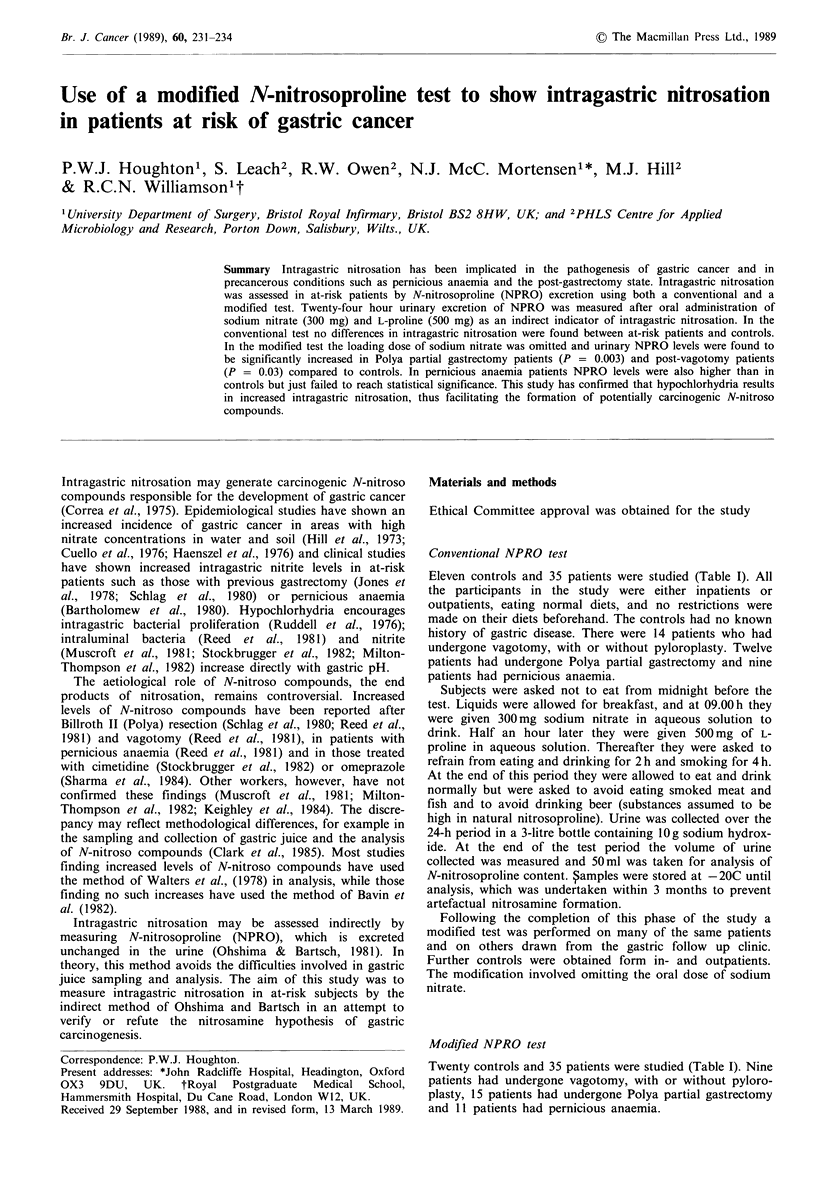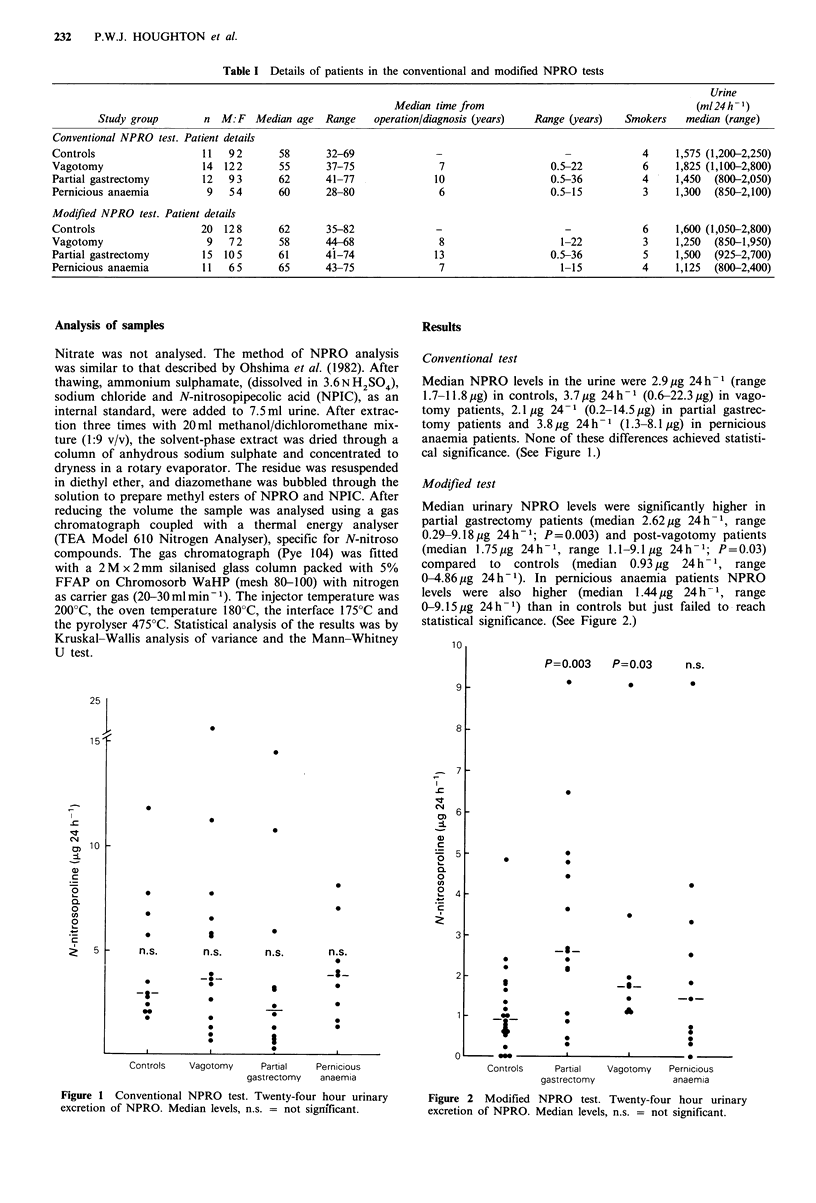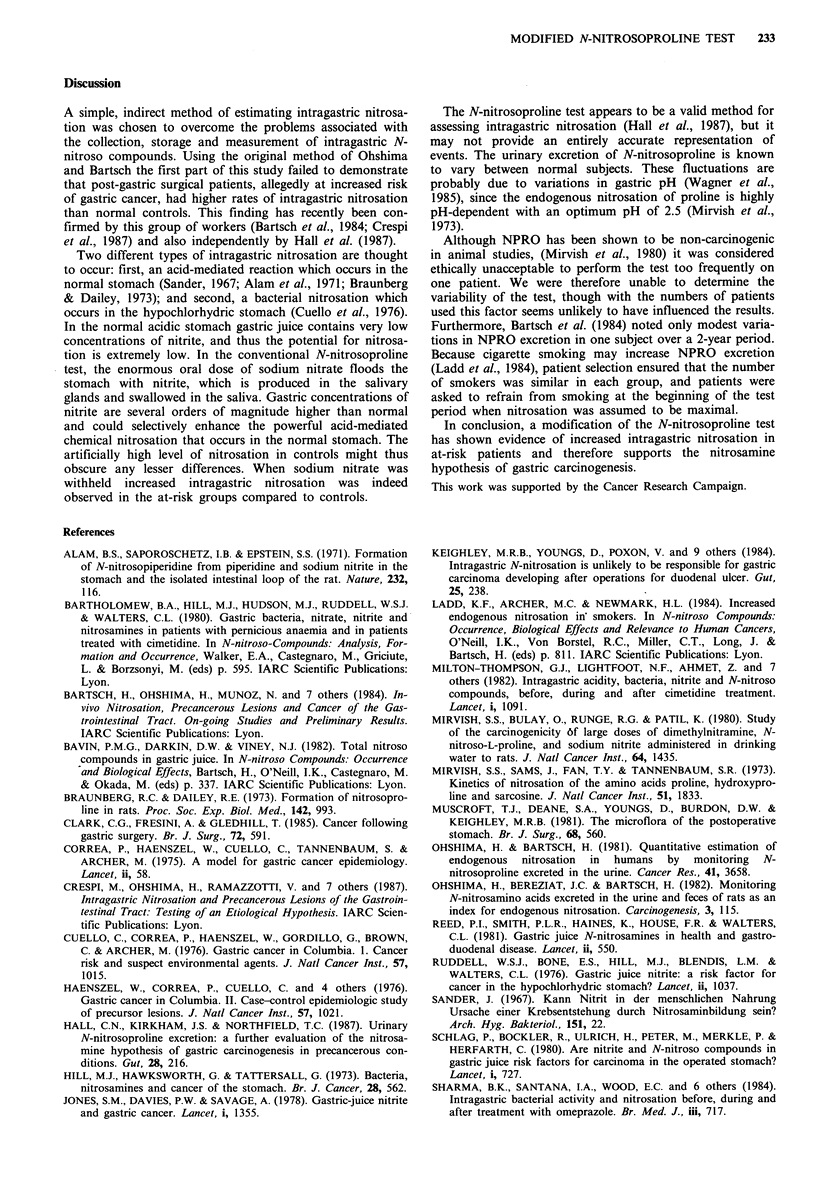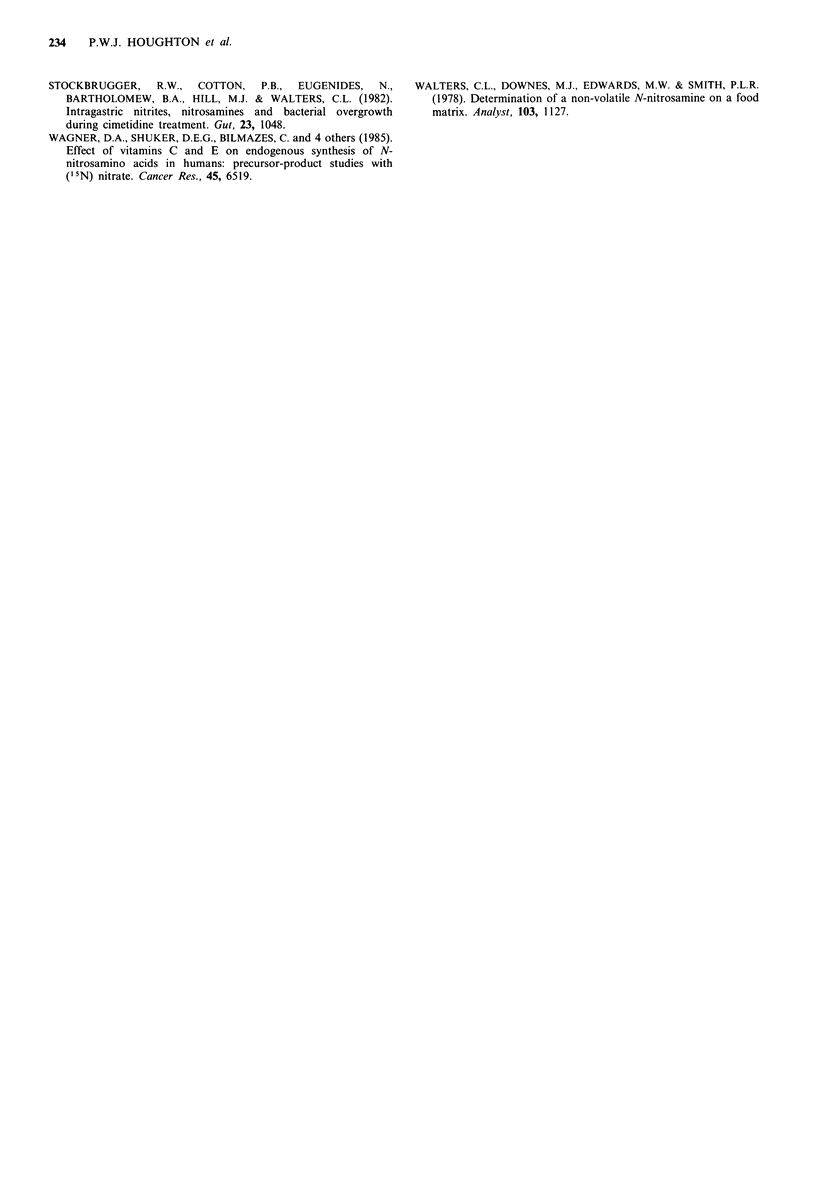# Use of a modified N-nitrosoproline test to show intragastric nitrosation in patients at risk of gastric cancer.

**DOI:** 10.1038/bjc.1989.258

**Published:** 1989-08

**Authors:** P. W. Houghton, S. Leach, R. W. Owen, N. J. McC Mortensen, M. J. Hill, R. C. Williamson

**Affiliations:** University Department of Surgery, Bristol Royal Infirmary, UK.

## Abstract

Intragastric nitrosation has been implicated in the pathogenesis of gastric cancer and in precancerous conditions such as pernicious anaemia and the post-gastrectomy state. Intragastric nitrosation was assessed in at-risk patients by N-nitrosoproline (NPRO) excretion using both a conventional and a modified test. Twenty-four hour urinary excretion of NPRO was measured after oral administration of sodium nitrate (300 mg) and L-proline (500 mg) as an indirect indicator of intragastric nitrosation. In the conventional test no differences in intragastric nitrosation were found between at-risk patients and controls. In the modified test the loading dose of sodium nitrate was omitted and urinary NPRO levels were found to be significantly increased in Polya partial gastrectomy patients (P = 0.003) and post-vagotomy patients (P = 0.03) compared to controls. In pernicious anaemia patients NPRO levels were also higher than in controls but just failed to reach statistical significance. This study has confirmed that hypochlorhydria results in increased intragastric nitrosation, thus facilitating the formation of potentially carcinogenic N-nitroso compounds.


Br. J. Cancer (1989), 60, 231–234 © The Macmillan Press Ltd., 1989

### Use of a modified *N*-nitrosoproline test to show intragastric nitrosation in patients at risk of gastric cancer

P.W.J. Houghton[1], S. Leach[2], R.W. Owen[2], N.J. McC. Mortensen[1]*, M.J. Hill[2] & R.C.N. Williamson[1]†

[1]*University Department of Surgery, Bristol Royal Infirmary, Bristol BS2 8HW, UK; and* [2]*PHLS Centre for Applied Microbiology and Research, Porton Down, Salisbury, Wilts., UK.*

**Summary** Intragastric nitrosation has been implicated in the pathogenesis of gastric cancer and in precancerous conditions such as pernicious anaemia and the post-gastrectomy state. Intragastric nitrosation was assessed in at-risk patients by *N*-nitrosoproline (NPRO) excretion using both a conventional and a modified test. Twenty-four hour urinary excretion of NPRO was measured after oral administration of sodium nitrate (300 mg) and L-proline (500 mg) as an indirect indicator of intragastric nitrosation. In the conventional test no differences in intragastric nitrosation were found between at-risk patients and controls. In the modified test the loading dose of sodium nitrate was omitted and urinary NPRO levels were found to be significantly increased in Polya partial gastrectomy patients ($P = 0.003$) and post-vagotomy patients ($P = 0.03$) compared to controls. In pernicious anaemia patients NPRO levels were also higher than in controls but just failed to reach statistical significance. This study has confirmed that hypochlorhydria results in increased intragastric nitrosation, thus facilitating the formation of potentially carcinogenic *N*-nitroso compounds.

Intragastric nitrosation may generate carcinogenic *N*-nitroso compounds responsible for the development of gastric cancer (Correa *et al.*, 1975). Epidemiological studies have shown an increased incidence of gastric cancer in areas with high nitrate concentrations in water and soil (Hill *et al.*, 1973; Cuello *et al.*, 1976; Haenszel *et al.*, 1976) and clinical studies have shown increased intragastric nitrite levels in at-risk patients such as those with previous gastrectomy (Jones *et al.*, 1978; Schlag *et al.*, 1980) or pernicious anaemia (Bartholomew *et al.*, 1980). Hypochlorhydria encourages intragastric bacterial proliferation (Ruddell *et al.*, 1976); intraluminal bacteria (Reed *et al.*, 1981) and nitrite (Muscroft *et al.*, 1981; Stockbrugger *et al.*, 1982; Milton-Thompson *et al.*, 1982) increase directly with gastric pH.

The aetiological role of *N*-nitroso compounds, the end products of nitrosation, remains controversial. Increased levels of *N*-nitroso compounds have been reported after Billroth II (Polya) resection (Schlag *et al.*, 1980; Reed *et al.*, 1981) and vagotomy (Reed *et al.*, 1981), in patients with pernicious anaemia (Reed *et al.*, 1981) and in those treated with cimetidine (Stockbrugger *et al.*, 1982) or omeprazole (Sharma *et al.*, 1984). Other workers, however, have not confirmed these findings (Muscroft *et al.*, 1981; Milton-Thompson *et al.*, 1982; Keighley *et al.*, 1984). The discrepancy may reflect methodological differences, for example in the sampling and collection of gastric juice and the analysis of *N*-nitroso compounds (Clark *et al.*, 1985). Most studies finding increased levels of *N*-nitroso compounds have used the method of Walters *et al.*, (1978) in analysis, while those finding no such increases have used the method of Bavin *et al.* (1982).

Intragastric nitrosation may be assessed indirectly by measuring *N*-nitrosoproline (NPRO), which is excreted unchanged in the urine (Ohshima & Bartsch, 1981). In theory, this method avoids the difficulties involved in gastric juice sampling and analysis. The aim of this study was to measure intragastric nitrosation in at-risk subjects by the indirect method of Ohshima and Bartsch in an attempt to verify or refute the nitrosamine hypothesis of gastric carcinogenesis.

Correspondence: P.W.J. Houghton.
Present addresses: *John Radcliffe Hospital, Headington, Oxford OX3 9DU, UK. †Royal Postgraduate Medical School, Hammersmith Hospital, Du Cane Road, London W12, UK.
Received 29 September 1988, and in revised form, 13 March 1989.

### Materials and methods

Ethical Committee approval was obtained for the study

### *Conventional NPRO test*

Eleven controls and 35 patients were studied (Table I). All the participants in the study were either inpatients or outpatients, eating normal diets, and no restrictions were made on their diets beforehand. The controls had no known history of gastric disease. There were 14 patients who had undergone vagotomy, with or without pyloroplasty. Twelve patients had undergone Polya partial gastrectomy and nine patients had pernicious anaemia.

Subjects were asked not to eat from midnight before the test. Liquids were allowed for breakfast, and at 09.00 h they were given 300 mg sodium nitrate in aqueous solution to drink. Half an hour later they were given 500 mg of L-proline in aqueous solution. Thereafter they were asked to refrain from eating and drinking for 2 h and smoking for 4 h. At the end of this period they were allowed to eat and drink normally but were asked to avoid eating smoked meat and fish and to avoid drinking beer (substances assumed to be high in natural nitrosoproline). Urine was collected over the 24-h period in a 3-litre bottle containing 10 g sodium hydroxide. At the end of the test period the volume of urine collected was measured and 50 ml was taken for analysis of *N*-nitrosoproline content. Samples were stored at −20C until analysis, which was undertaken within 3 months to prevent artefactual nitrosamine formation.

Following the completion of this phase of the study a modified test was performed on many of the same patients and on others drawn from the gastric follow up clinic. Further controls were obtained form in- and outpatients. The modification involved omitting the oral dose of sodium nitrate.

### *Modified NPRO test*

Twenty controls and 35 patients were studied (Table I). Nine patients had undergone vagotomy, with or without pyloroplasty, 15 patients had undergone Polya partial gastrectomy and 11 patients had pernicious anaemia.

**Table I** Details of patients in the conventional and modified NPRO tests

| *Study group* | *n* | *M:F* | *Median age* | *Range* | *Median time from operation/diagnosis (years)* | *Range (years)* | *Smokers* | *Urine ($ml\,24\,h^{-1}$) median (range)* |
|---|---|---|---|---|---|---|---|---|
| *Conventional NPRO test. Patient details* | | | | | | | | |
| Controls | 11 | 9 2 | 58 | 32–69 | – | – | 4 | 1,575 (1,200–2,250) |
| Vagotomy | 14 | 12 2 | 55 | 37–75 | 7 | 0.5–22 | 6 | 1,825 (1,100–2,800) |
| Partial gastrectomy | 12 | 9 3 | 62 | 41–77 | 10 | 0.5–36 | 4 | 1,450 (800–2,050) |
| Pernicious anaemia | 9 | 5 4 | 60 | 28–80 | 6 | 0.5–15 | 3 | 1,300 (850–2,100) |
| *Modified NPRO test. Patient details* | | | | | | | | |
| Controls | 20 | 12 8 | 62 | 35–82 | – | – | 6 | 1,600 (1,050–2,800) |
| Vagotomy | 9 | 7 2 | 58 | 44–68 | 8 | 1–22 | 3 | 1,250 (850–1,950) |
| Partial gastrectomy | 15 | 10 5 | 61 | 41–74 | 13 | 0.5–36 | 5 | 1,500 (925–2,700) |
| Pernicious anaemia | 11 | 6 5 | 65 | 43–75 | 7 | 1–15 | 4 | 1,125 (800–2,400) |

### Analysis of samples

Nitrate was not analysed. The method of NPRO analysis was similar to that described by Ohshima *et al.* (1982). After thawing, ammonium sulphamate, (dissolved in 3.6 N $H_2SO_4$), sodium chloride and *N*-nitrosopipecolic acid (NPIC), as an internal standard, were added to 7.5 ml urine. After extraction three times with 20 ml methanol/dichloromethane mixture (1:9 v/v), the solvent-phase extract was dried through a column of anhydrous sodium sulphate and concentrated to dryness in a rotary evaporator. The residue was resuspended in diethyl ether, and diazomethane was bubbled through the solution to prepare methyl esters of NPRO and NPIC. After reducing the volume the sample was analysed using a gas chromatograph coupled with a thermal energy analyser (TEA Model 610 Nitrogen Analyser), specific for *N*-nitroso compounds. The gas chromatograph (Pye 104) was fitted with a 2 M × 2 mm silanised glass column packed with 5% FFAP on Chromosorb WaHP (mesh 80–100) with nitrogen as carrier gas (20–30 ml $min^{-1}$). The injector temperature was 200°C, the oven temperature 180°C, the interface 175°C and the pyrolyser 475°C. Statistical analysis of the results was by Kruskal–Wallis analysis of variance and the Mann–Whitney U test.

**Figure 1** Conventional NPRO test. Twenty-four hour urinary excretion of NPRO. Median levels, n.s. = not significant.

### Results

### *Conventional test*

Median NPRO levels in the urine were 2.9 μg 24 h$^{-1}$ (range 1.7–11.8 μg) in controls, 3.7 μg 24 h$^{-1}$ (0.6–22.3 μg) in vagotomy patients, 2.1 μg 24$^{-1}$ (0.2–14.5 μg) in partial gastrectomy patients and 3.8 μg 24 h$^{-1}$ (1.3–8.1 μg) in pernicious anaemia patients. None of these differences achieved statistical significance. (See Figure 1.)

### *Modified test*

Median urinary NPRO levels were significantly higher in partial gastrectomy patients (median 2.62 μg 24 h$^{-1}$, range 0.29–9.18 μg 24 h$^{-1}$; $P$=0.003) and post-vagotomy patients (median 1.75 μg 24 h$^{-1}$, range 1.1–9.1 μg 24 h$^{-1}$; $P$=0.03) compared to controls (median 0.93 μg 24 h$^{-1}$, range 0–4.86 μg 24 h$^{-1}$). In pernicious anaemia patients NPRO levels were also higher (median 1.44 μg 24 h$^{-1}$, range 0–9.15 μg 24 h$^{-1}$) than in controls but just failed to reach statistical significance. (See Figure 2.)

**Figure 2** Modified NPRO test. Twenty-four hour urinary excretion of NPRO. Median levels, n.s. = not significant.

### Discussion

A simple, indirect method of estimating intragastric nitrosation was chosen to overcome the problems associated with the collection, storage and measurement of intragastric *N*-nitroso compounds. Using the original method of Ohshima and Bartsch the first part of this study failed to demonstrate that post-gastric surgical patients, allegedly at increased risk of gastric cancer, had higher rates of intragastric nitrosation than normal controls. This finding has recently been confirmed by this group of workers (Bartsch *et al.*, 1984; Crespi *et al.*, 1987) and also independently by Hall *et al.* (1987).

Two different types of intragastric nitrosation are thought to occur: first, an acid-mediated reaction which occurs in the normal stomach (Sander, 1967; Alam *et al.*, 1971; Braunberg & Dailey, 1973); and second, a bacterial nitrosation which occurs in the hypochlorhydric stomach (Cuello *et al.*, 1976). In the normal acidic stomach gastric juice contains very low concentrations of nitrite, and thus the potential for nitrosation is extremely low. In the conventional *N*-nitrosoproline test, the enormous oral dose of sodium nitrate floods the stomach with nitrite, which is produced in the salivary glands and swallowed in the saliva. Gastric concentrations of nitrite are several orders of magnitude higher than normal and could selectively enhance the powerful acid-mediated chemical nitrosation that occurs in the normal stomach. The artificially high level of nitrosation in controls might thus obscure any lesser differences. When sodium nitrate was withheld increased intragastric nitrosation was indeed observed in the at-risk groups compared to controls.

The *N*-nitrosoproline test appears to be a valid method for assessing intragastric nitrosation (Hall *et al.*, 1987), but it may not provide an entirely accurate representation of events. The urinary excretion of *N*-nitrosoproline is known to vary between normal subjects. These fluctuations are probably due to variations in gastric pH (Wagner *et al.*, 1985), since the endogenous nitrosation of proline is highly pH-dependent with an optimum pH of 2.5 (Mirvish *et al.*, 1973).

Although NPRO has been shown to be non-carcinogenic in animal studies, (Mirvish *et al.*, 1980) it was considered ethically unacceptable to perform the test too frequently on one patient. We were therefore unable to determine the variability of the test, though with the numbers of patients used this factor seems unlikely to have influenced the results. Furthermore, Bartsch *et al.* (1984) noted only modest variations in NPRO excretion in one subject over a 2-year period. Because cigarette smoking may increase NPRO excretion (Ladd *et al.*, 1984), patient selection ensured that the number of smokers was similar in each group, and patients were asked to refrain from smoking at the beginning of the test period when nitrosation was assumed to be maximal.

In conclusion, a modification of the *N*-nitrosoproline test has shown evidence of increased intragastric nitrosation in at-risk patients and therefore supports the nitrosamine hypothesis of gastric carcinogenesis.

This work was supported by the Cancer Research Campaign.

### References

ALAM, B.S., SAPOROSCHETZ, I.B. & EPSTEIN, S.S. (1971). Formation of *N*-nitrosopiperidine from piperidine and sodium nitrite in the stomach and the isolated intestinal loop of the rat. *Nature*, **232**, 116.

BARTHOLOMEW, B.A., HILL, M.J., HUDSON, M.J., RUDDELL, W.S.J. & WALTERS, C.L. (1980). Gastric bacteria, nitrate, nitrite and nitrosamines in patients with pernicious anaemia and in patients treated with cimetidine. In *N-nitroso-Compounds: Analysis, Formation and Occurrence*, Walker, E.A., Castegnaro, M., Griciute, L. & Borzsonyi, M. (eds) p. 595. IARC Scientific Publications: Lyon.

BARTSCH, H., OHSHIMA, H., MUNOZ, N. and 7 others (1984). *Invivo Nitrosation, Precancerous Lesions and Cancer of the Gastrointestinal Tract. On-going Studies and Preliminary Results.* IARC Scientific Publications: Lyon.

BAVIN, P.M.G., DARKIN, D.W. & VINEY, N.J. (1982). Total nitroso compounds in gastric juice. In *N-nitroso Compounds: Occurrence and Biological Effects*, Bartsch, H., O'Neill, I.K., Castegnaro, M. & Okada, M. (eds) p. 337. IARC Scientific Publications: Lyon.

BRAUNBERG, R.C. & DAILEY, R.E. (1973). Formation of nitrosoproline in rats. *Proc. Soc. Exp. Biol. Med.*, **142**, 993.

CLARK, C.G., FRESINI, A. & GLEDHILL, T. (1985). Cancer following gastric surgery. *Br. J. Surg.*, **72**, 591.

CORREA, P., HAENSZEL, W., CUELLO, C., TANNENBAUM, S. & ARCHER, M. (1975). A model for gastric cancer epidemiology. *Lancet*, **ii**, 58.

CRESPI, M., OHSHIMA, H., RAMAZZOTTI, V. and 7 others (1987). *Intragastric Nitrosation and Precancerous Lesions of the Gastrointestinal Tract: Testing of an Etiological Hypothesis.* IARC Scientific Publications: Lyon.

CUELLO, C., CORREA, P., HAENSZEL, W., GORDILLO, G., BROWN, C. & ARCHER, M. (1976). Gastric cancer in Columbia. 1. Cancer risk and suspect environmental agents. *J. Natl Cancer Inst.*, **57**, 1015.

HAENSZEL, W., CORREA, P., CUELLO, C. and 4 others (1976). Gastric cancer in Columbia. II. Case–control epidemiologic study of precursor lesions. *J. Natl Cancer Inst.*, **57**, 1021.

HALL, C.N., KIRKHAM, J.S. & NORTHFIELD, T.C. (1987). Urinary *N*-nitrosoproline excretion: a further evaluation of the nitrosamine hypothesis of gastric carcinogenesis in precancerous conditions. *Gut*, **28**, 216.

HILL, M.J., HAWKSWORTH, G. & TATTERSALL, G. (1973). Bacteria, nitrosamines and cancer of the stomach. *Br. J. Cancer*, **28**, 562.

JONES, S.M., DAVIES, P.W. & SAVAGE, A. (1978). Gastric-juice nitrite and gastric cancer. *Lancet*, **i**, 1355.

KEIGHLEY, M.R.B., YOUNGS, D., POXON, V. and 9 others (1984). Intragastric *N*-nitrosation is unlikely to be responsible for gastric carcinoma developing after operations for duodenal ulcer. *Gut*, **25**, 238.

LADD, K.F., ARCHER, M.C. & NEWMARK, H.L. (1984). Increased endogenous nitrosation in smokers. In *N-nitroso Compounds: Occurrence, Biological Effects and Relevance to Human Cancers*, O'Neill, I.K., Von Borstel, R.C., Miller, C.T., Long, J. & Bartsch, H. (eds) p. 811. IARC Scientific Publications: Lyon.

MILTON-THOMPSON, G.J., LIGHTFOOT, N.F., AHMET, Z. and 7 others (1982). Intragastric acidity, bacteria, nitrite and *N*-nitroso compounds, before, during and after cimetidine treatment. *Lancet*, **i**, 1091.

MIRVISH, S.S., BULAY, O., RUNGE, R.G. & PATIL, K. (1980). Study of the carcinogenicity of large doses of dimethylnitramine, *N*-nitroso-L-proline, and sodium nitrite administered in drinking water to rats. *J. Natl Cancer Inst.*, **64**, 1435.

MIRVISH, S.S., SAMS, J., FAN, T.Y. & TANNENBAUM, S.R. (1973). Kinetics of nitrosation of the amino acids proline, hydroxyproline and sarcosine. *J. Natl Cancer Inst.*, **51**, 1833.

MUSCROFT, T.J., DEANE, S.A., YOUNGS, D., BURDON, D.W. & KEIGHLEY, M.R.B. (1981). The microflora of the postoperative stomach. *Br. J. Surg.*, **68**, 560.

OHSHIMA, H. & BARTSCH, H. (1981). Quantitative estimation of endogenous nitrosation in humans by monitoring *N*-nitrosoproline excreted in the urine. *Cancer Res.*, **41**, 3658.

OHSHIMA, H., BEREZIAT, J.C. & BARTSCH, H. (1982). Monitoring *N*-nitrosamino acids excreted in the urine and feces of rats as an index for endogenous nitrosation. *Carcinogenesis*, **3**, 115.

REED, P.I., SMITH, P.L.R., HAINES, K., HOUSE, F.R. & WALTERS, C.L. (1981). Gastric juice *N*-nitrosamines in health and gastroduodenal disease. *Lancet*, **ii**, 550.

RUDDELL, W.S.J., BONE, E.S., HILL, M.J., BLENDIS, L.M. & WALTERS, C.L. (1976). Gastric juice nitrite: a risk factor for cancer in the hypochlorhydric stomach? *Lancet*, **ii**, 1037.

SANDER, J. (1967). Kann Nitrit in der menschlichen Nahrung Ursache einer Krebsentstehung durch Nitrosaminbildung sein? *Arch. Hyg. Bakteriol.*, **151**, 22.

SCHLAG, P., BOCKLER, R., ULRICH, H., PETER, M., MERKLE, P. & HERFARTH, C. (1980). Are nitrite and *N*-nitroso compounds in gastric juice risk factors for carcinoma in the operated stomach? *Lancet*, **i**, 727.

SHARMA, B.K., SANTANA, I.A., WOOD, E.C. and 6 others (1984). Intragastric bacterial activity and nitrosation before, during and after treatment with omeprazole. *Br. Med. J.*, **iii**, 717.

STOCKBRUGGER, R.W., COTTON, P.B., EUGENIDES, N., BARTHOLOMEW, B.A., HILL, M.J. & WALTERS, C.L. (1982). Intragastric nitrites, nitrosamines and bacterial overgrowth during cimetidine treatment. *Gut*, **23**, 1048.

WAGNER, D.A., SHUKER, D.E.G., BILMAZES, C. and 4 others (1985). Effect of vitamins C and E on endogenous synthesis of *N*-nitrosamino acids in humans: precursor-product studies with ($^{15}N$) nitrate. *Cancer Res.*, **45**, 6519.

WALTERS, C.L., DOWNES, M.J., EDWARDS, M.W. & SMITH, P.L.R. (1978). Determination of a non-volatile *N*-nitrosamine on a food matrix. *Analyst*, **103**, 1127.